# Comparison of prognostic impact of anticoagulants in heart failure patients with atrial fibrillation and renal dysfunction: direct oral anticoagulants versus vitamin K antagonists

**DOI:** 10.1007/s00380-022-02027-w

**Published:** 2022-01-21

**Authors:** Takahiro Sakai, Hirohiko Motoki, Aya Fuchida, Takahiro Takeuchi, Kyuhachi Otagiri, Masafumi Kanai, Kazuhiro Kimura, Masatoshi Minamisawa, Koji Yoshie, Tatsuya Saigusa, Soichiro Ebisawa, Ayako Okada, Hiroshi Kitabayashi, Koichiro Kuwahara

**Affiliations:** 1 Department of Cardiovascular Medicine, Ina Central Hospital, Ina, Japan; 2 Department of Cardiovascular Medicine, Shinshu University School of Medicine, 3-1-1 Asahi, Matsumoto, 390-8621 Japan

**Keywords:** Vitamin K antagonist, Direct oral anticoagulants, Atrial fibrillation, Heart failure, Renal dysfunction

## Abstract

Although high thromboembolic risk was assumed in elderly patients with heart failure (HF) and atrial fibrillation (AF), inadequate control of prothrombin time/international normalized ratio was often observed in patients using vitamin K antagonists (VKAs). We hypothesized that patients treated with direct oral anticoagulants (DOAC) would have a better outcome than those treated with VKAs. The aim of this study was to compare the efficacies of DOACs and VKAs in elderly patients with HF and AF. We retrospectively analyzed data from a multicenter, prospective observational cohort study. A total of 1036 patients who were hospitalized for acute decompensated HF were enrolled. We assessed 329 patients aged > 65 years who had non-valvular AF and divided them into 2 groups according to the anticoagulant therapy they received. A subgroup analysis was performed using renal dysfunction based on estimated glomerular filtration rate (eGFR; mL/min/1.73 m^2^). The primary outcome was all-cause mortality, and the secondary outcomes were non-cardiovascular death or stroke. The median follow-up period was 730 days (range 334–1194 days). The primary outcome was observed in 84 patients; non-cardiovascular death, in 25 patients; and stroke, in 14 patients. The Kaplan–Meier analysis revealed that all-cause mortality was significantly lower in the DOAC group than in the VKA group (log-rank *p* = 0.033), whereas the incidence rates of non-cardiovascular death (log-rank *p* = 0.171) and stroke (log-rank *p* = 0.703) were not significantly different in the crude population. DOAC therapy was not associated with lower mortality in the crude population (log-rank *p* = 0.146) and in the eGFR ≥ 45 mL/min/1.73 m^2^ subgroup (log-rank *p* = 0.580). However, DOAC therapy was independently associated with lower mortality after adjustments for age, diabetes mellitus, and albumin level (hazard ratio, 0.55; 95% confidence interval, 0.30–0.99; *p* = 0.045) in the eGFR < 45 mL/min/1.73 m^2^ subgroup. Compared with VKA therapy, DOAC therapy was associated with lower risk of all-cause mortality in the elderly HF patients with AF and renal dysfunction.

## Introduction

Atrial fibrillation (AF) is a common form of cardiac arrhythmia in elderly patients. Moreover, heart failure (HF) or renal dysfunction frequently coexists with AF [1], and high thromboembolic risk was assumed in elderly patients with HF and AF [2]. Vitamin K antagonists (VKA) have been the standard therapeutic agents for patients with AF for decades, and a previous study reported that VKA reduced stroke risk by 64% and mortality by 26% as compared with placebo [3]. VKAs are effective and relatively safe drugs with an adequate time in therapeutic range (TTR) of > 70%, but inadequate control of prothrombin time/international normalized ratio is often observed in patients using VKAs [4]. A subanalysis of data from the ROCKET AF (Rivaroxaban Once Daily Oral Direct Factor Xa Inhibitor Compared with Vitamin K Antagonism for Prevention of Stroke and Embolism Trial in Atrial Fibrillation) trial reported that renal dysfunction was associated with a lower TTR of VKA during the administration period [5]. In the Outcomes Registry for Better Informed Treatment of Atrial Fibrillation registry, multiple comorbidities such as frailty, HF, renal dysfunction, chronic obstructive pulmonary disease, and diabetes were identified as risk factors of lower TTR [6]. Patients with multiple comorbidities tend to be given more concomitant drugs, which are associated with low treatment adherence. Direct oral anticoagulants (DOACs) have become common therapeutic agents for preventing stroke or systemic embolism in patients with AF. Four DOACs are available for paroxysmal/persistent AF. Previous studies revealed that use of DOACs as compared with VKAs was associated with lower risks of stroke, systemic embolism, and major bleeding [7, 8], but the efficacy of DOACs in patients with renal dysfunction has not been fully discussed. Physicians sometimes hesitate to prescribe DOACs for elderly patients with renal dysfunction because of fear of hemorrhagic complications. Recently, Makani et al. [9] reported the safety and efficacy of DOAC use in patients with concomitant renal dysfunction and AF. However, a meta-analysis of DOACs indicated that the standard dose of DOACs as compared with that of VKAs was safer and more effective in Asians than in non-Asians [10]. We hypothesized that better outcomes can be obtained using DOACs in high-risk patients with HF. The aim of this study was to evaluate the efficacy of DOACs in HF patients with AF and renal dysfunction in the Japanese population.

## Materials and methods

### Patient population

This study was a post hoc analysis of a multicenter, prospective observational cohort study. In this study, 1036 consecutive patients who were admitted at 13 institutions in Nagano Prefecture, Japan, between July 2014 and December 2018 because of acute decompensated HF (ADHF) were enrolled. The diagnosis of HF was based on the criteria used in the Framingham study [11]. The exclusion criteria were patients aged < 20 years, those who were impossible to follow-up, those from whom informed consent was difficult to obtain, and those with acute coronary syndrome. After admission, medical therapy was initiated at the discretion of the physician. Clinical data, including patient demographics, past medical history, drug usage, echocardiography findings, electrocardiography findings, and laboratory data, were collected during the compensated state of HF. Echocardiography was performed in accordance with the recommendations of the American Society of Echocardiography [12]. After the patients’ discharge, follow-up data were collected from their medical records or through telephone interview. The clinical events were all-cause death, cardiovascular death, acute coronary syndrome, stroke, hospitalization for acute decompensated HF, and hospitalization for any cardiovascular disease. This study was approved by the ethics committees of the hospitals and performed in accordance with the tenets of the 1975 Declaration of Helsinki for clinical research protocols. Informed consent was obtained from all the patients.

### Study protocol

**Fig. 1:**
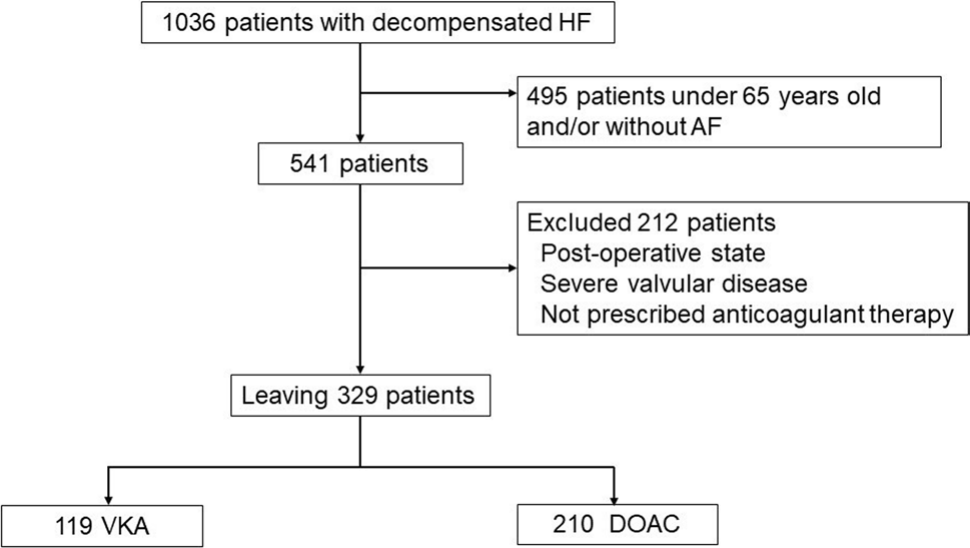
Study flow diagram. *AF* atrial fibrillation, *DOAC* direct oral anticoagulant, *HF* heart failure, *VKA* vitamin K antagonists

We screened 541 patients aged > 65 years who had paroxysmal/persistent AF and excluded 212 patients who had undergone surgery, had severe valvular disease, and had not received anticoagulant therapy. The remaining 329 patients were assessed as having non-valvular AF (NVAF) and divided into 2 groups according to the anticoagulant therapy they received (Fig. 1). A subgroup analysis was performed using renal dysfunction based on estimated glomerular filtration rate (eGFR; mL/min/1.73 m^2^). The cutoff value was set at 45 mL/min/1.73 m^2^ as an approximate value of median eGFR (46 mL/min/1.73m^2^) and as the boundary of chronic kidney disease (CKD) grade between G3a and G3b. The primary outcome was all-cause mortality, and the secondary outcomes were non-cardiovascular death and stroke.

### Statistical analyses

Continuous variables are expressed as mean ± standard deviation when normally distributed and as median and interquartile range when non-normally distributed. Normality was assessed using the Shapiro–Wilk *W* test. Categorical variables are expressed as number and percentage. The patients’ baseline characteristics were compared using a contingency table and *t* test for normally distributed continuous variables, the Mann–Whitney *U* test for non-normally distributed continuous variables, and the chi-square test for categorical variables. *P* values of < 0.05 were considered statistically significant. The cumulative incidence of the end point was analyzed using the Kaplan–Meier method, and differences were assessed with the log-rank test. Cox proportional hazard analysis was performed to adjust for baseline differences and assess whether the variables were independent predictors of outcome. Following univariate analysis, covariates that were associated with outcome (*p* < 0.1) were included in the multivariate model. The SPSS version 27.0 statistical software (SPSS Inc., Chicago, Illinois) was used in the analysis.

## Results

### Patients’ characteristics

Among the 329 patients, 119 were treated with VKA and 210 were treated with DOACs. The patients’ baseline characteristics are shown in Table 1. The median age was 82 years (interquartile range: 76–86 years), and 49% (*n* = 161) of the patients were female. Among the comorbidities, diabetes mellitus was more frequently observed in the VKA group, but history of stroke was more prevalent in the DOAC group. Laboratory data indicated that hemoglobin level was lower in the VKA group, but serum albumin level, eGFR, brain natriuretic peptide level, and C-reactive protein level were not significantly different between the two groups. Whereas β-blocker was more frequently prescribed in the DOAC group, the use frequencies of antiplatelet and conventional drugs for HF, including angiotensin-converting enzyme inhibitor or angiotensin receptor blocker, and mineralocorticoid receptor antagonist were not markedly different between the two groups.

**Table 1:** Baseline characteristics of the crude population and subgroups

	Crude population *n* = 329			eGFR < 45 (mL/min/1.73 m^2^) *n* = 159			eGFR ≥ 45 (mL/min/1.73 m^2^) *n* = 170		
	VKA * n* = 119	DOAC * n* = 210	p value	VKA * n* = 65	DOAC * n* = 94	p value	VKA * n* = 54	DOAC * n* = 116	*p* value
Age (years)	82 (76, 86)	83 (76, 87)	0.867	83 (79, 88)	84 (80, 88)	0.381	81 (72, 85)	79 (73, 86)	0.830
Female, *n* (%)	57 (48.3%)	104 (49.3%)	0.911	33 (50.8%)	46 (48.9%)	0.823	24 (44.4%)	58 (50.0%)	0.501
Body mass index (kg/m^2^)	20.2 (18.8, 23.5)	21.4 (19.1, 24.2)	0.111	20.5 (19.2, 24.5)	21.8 (19.1, 24.7)	0.788	19.6 (18.2, 22.4)	21.1 (19.1, 24.0)	0.023
Heart rate (beats per minute)	70 (60, 80)	72 (63, 83)	0.062	66 (60, 77)	73 (63, 85)	0.018	75 (60, 82)	72 (62, 82)	0.883
Systolic blood pressure (mmHg)	112 ± 17	113 ± 16	0.690	114 ± 17	110 ± 17	0.950	107 ± 17	111 ± 17	0.430
Hypertension, *n* (%)	80 (66.7%)	136 (65.1%)	0.774	50 (76.9%)	65 (69.1%)	0.332	29 (53.7%)	71 (61.2%)	0.358
Dyslipidemia, *n* (%)	29 (24.2%)	52 (24.9%)	0.880	18 (27.7%)	27 (28.7%)	0.847	11 (20.3%)	25 (21.6%)	0.861
Diabetes mellitus, *n* (%)	40 (33.3%)	48 (23.0%)	0.041	20 (30.8%)	22 (23.4%)	0.324	19 (35.2%)	26 (22.4%)	0.079
Hyperuricemia, *n* (%)	26 (21.7%)	40 (19.1%)	0.583	19 (29.2%)	25 (26.6%)	0.745	6 (11.1%)	15 (12.9%)	0.737
COPD, *n* (%)	8 (6.7%)	11 (5.3%)	0.604	2 (3.1%)	4 (4.3%)	0.688	6 (11.1%)	7 (6.0%)	0.246
Hemodialysis, *n* (%)	0 (0%)	0 (0%)	–	0 (0%)	0 (0%)	–	0 (0%)	0 (0%)	–
Coronary artery disease, *n* (%)	24 (20.0%)	39 (18.7%)	0.782	12 (18.5%)	22 (23.4%)	0.414	11 (20.4%)	17 (14.7%)	0.350
Stroke, *n* (%)	13 (10.8%)	40 (19.1%)	0.049	6 (9.2%)	20 (21.3%)	0.041	7 (13.0%)	20 (17.2%)	0.477
Neoplasm, *n* (%)	6 (5.0%)	20 (9.6%)	0.142	4 (6.2%)	9 (9.6%)	0.432	2 (3.7%)	11 (9.5%)	0.187
Laboratory data									
Albumin (g/dL)	3.4 ± 0.5	3.4 ± 0.5	0.627	3.3 ± 0.5	3.4 ± 0.4	0.490	3.4 ± 0.5	3.4 ± 0.5	0.805
Hemoglobin (g/dL)	11.4 (10.2, 13.1)	12.4 (11.0, 13.9)	< 0.001	11.0 (10.0, 12.4)	11.7 (10.6, 13.6)	0.044	12.1 (10.5, 13.8)	12.8 (11.5, 14.7)	0.011
eGFR (mL/min/1.73 m^2^)	43.9 (30.8, 56.8)	46.2 (36.0, 60.0)	0.057	33.0 (25.4, 39.0)	34.8 (29.7, 39.3)	0.105	58.0 (51.0, 68.0)	59.0 (50.0, 65.8)	0.624
BNP (pg/mL)	302 (144, 544)	258 (138, 451)	0.264	363 (166, 645)	304 (153, 497)	0.155	216 (117, 424)	224 (132, 409)	0.812
CRP (mg/dL)	0.33 (0.10, 0.69)	0.30 (0.10, 0.96)	0.675	0.19 (0.10, 0.67)	0.32 (0.10, 0.89)	0.424	0.39 (0.09, 0.74)	0.30 (0.10, 1.01)	0.784
PT-INR	1.89 ± 0.46	1.21 ± 0.14	< 0.001	1.77 ± 0.36	1.20 ± 0.12	< 0.001	2.07 ± 0.53	1.20 ± 0.17	< 0.001
CHADS2 score	3 (2, 4)	3 (2, 4)	0.981	3 (2, 4)	3 (2, 4)	0.862	3 (2,4)	3 (2, 4)	0.821
Medication									
Antiplatelet drugs, *n* (%)	33 (28.3%)	52 (24.9%)	0.499	17 (26.2%)	28 (29.8%)	0.607	16 (29.6%)	24 (20.7%)	0.350
ACEi/ARB, *n* (%)	83 (69.2%)	147 (70.3%)	0.826	40 (61.5%)	64 (68.0%)	0.394	42 (77.8%)	83 (71.6%)	0.392
Beta-blocker, *n* (%)	84 (70.0%)	167 (79.9%)	0.042	44 (67.7%)	75 (79.8%)	0.063	39 (72.2%)	92 (79.3%)	0.306
MRA, *n* (%)	60 (50.0%)	118 (56.4%)	0.268	30 (46.1%)	49 (52.1%)	0.459	30 (55.6%)	69 (59.5%)	0.629
SGLT2 inhibitor, *n* (%)	0 (0%)	7 (3.3%)	0.051	0 (0%)	2 (2.1%)	0.237	0 (0%)	5 (4.3%)	0.121

*ACEi* angiotensin converting enzyme inhibitor, *ARB* angiotensin receptor blocker, *BNP* brain natriuretic peptide, *COPD* chronic obstructive pulmonary disease, *CRP* C-reactive protein, *DOAC* direct oral anticoagulant, *eGFR* estimated glomerular filtration rate, *MRA* mineralocorticoid receptor antagonist, *SGLT* sodium glucose cotransporter, *VKA* vitamin K antagonist

### Outcomes

**Fig. 2:**
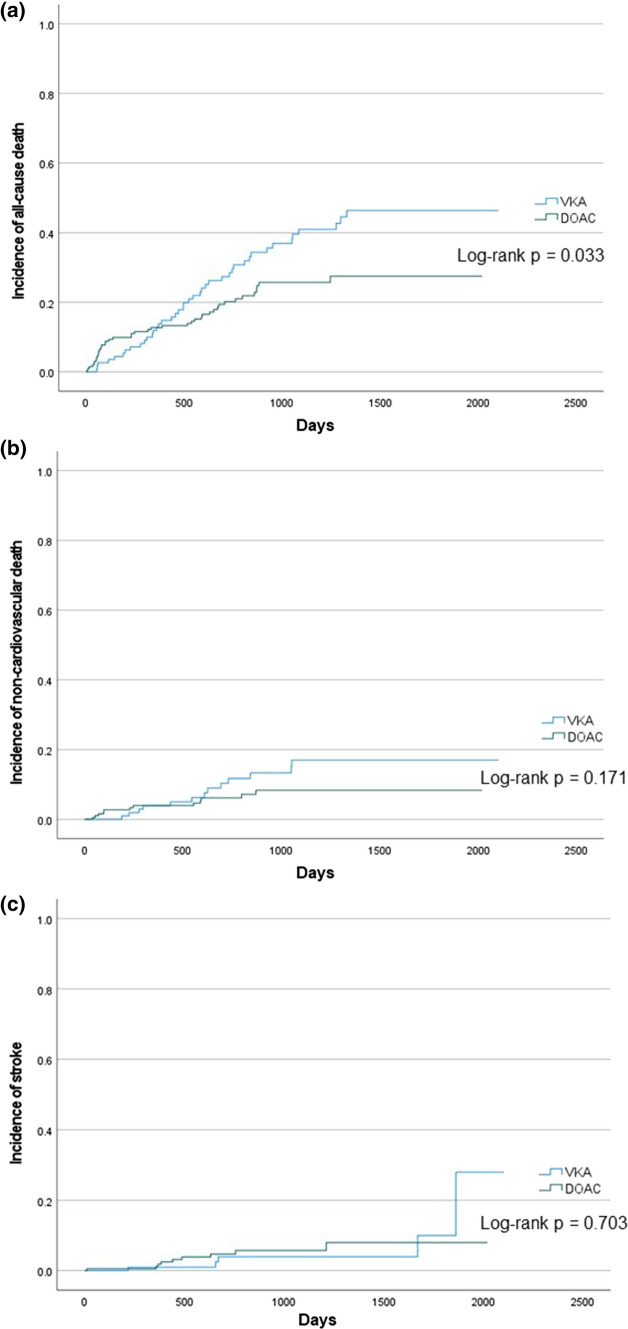
Kaplan–Meier analysis for the incidence of all-cause mortality, non-cardiovascular death, stroke. All-cause mortality was significantly lower in the DOAC group than in the VKA group, but non-cardiovascular death and stroke were not statistically significantly different between the two groups. *DOAC* direct oral anticoagulant, *VKA* vitamin K antagonists

The median follow-up period was 730 days (interquartile range 334–1194 days). The primary outcome was observed in 84 patients (25.5%). Non-cardiovascular death occurred in 25 patients (7.6%), and stroke occurred in 14 patients (4.3%). The hazard ratio (HRs) and 95% confidence interval (CIs) for the primary and secondary outcomes are shown in Table 2. The incidence of non-cardiovascular death and stroke were not statistically significantly different between the two groups, but all-cause death was more frequently observed in the VKA group. The Kaplan–Meier analyses for the primary and secondary outcomes are shown in Fig. 2. All-cause mortality was significantly lower in the DOAC group than in the VKA group (log-rank *p* = 0.033), while the incidence rates of non-cardiovascular death and stroke were not significantly different between the two groups. DOAC use was not significantly associated with all-cause mortality (HR, 0.71; 95% CI 0.45–1.13; *p* = 0.146) in multivariate analysis of the crude population, although it was associated with a lower incidence of the primary outcome (HR, 0.63; 95% CI 0.41–0.97; *p* = 0.035) in univariate analysis (Table 3).

**Table 2:** Incidence of primary and secondary outcomes

	All *n* = 329	VKA *n* = 119	DOAC *n* = 210	Crude HR (VKA vs. DOAC)	*p* value
All-cause death	84 (25.5%)	42 (35.0%)	42 (20.1%)	1.59 (1.03–2.43)	0.004
Non-cardiovascular death	25 (7.6%)	13 (10.8%)	12 (5.7%)	1.73 (0.79–3.79)	0.134
Stroke	14 (4.3%)	5 (4.2%)	9 (4.3%)	0.80 (0.27–2.43)	1.000
Hemorrhagic	2 (0.6%)	1 (0.8%)	1 (0.5%)	1.68 (0.11–26.9)	1.000
Ischemic	12 (3.6%)	4 (3.3%)	8 (3.8%)	0.70 (0.21–2.38)	1.000

*DOAC* direct oral anticoagulant, *HR* hazard ratio, *VKA* vitamin K antagonist

**Table 3:** Cox multivariate analysis for all-cause death in the crude population

	Crude population			
	Unadjusted HR (95% CI)	*p* value	Adjusted HR (95% CI)	*p* value
Age	1.07 (1.03–1.11)	< 0.001	1.04 (1.00–1.08)	0.044
Female	0.70 (0.45–1.08)	0.105		
BMI	0.90 (0.85–0.96)	0.001	0.92 (0.87–0.98)	0.010
Hypertension	0.86 (0.55–1.33)	0.494		
Dyslipidemia	0.93 (0.57–1.53)	0.782		
Diabetes mellitus	1.24 (0.78–1.96)	0.358		
Hyperuricemia	0.76 (0.50–1.32)	0.180		
COPD	2.22 (1.15–4.30)	0.018	2.24 (1.10–4.56)	0.026
CAD	1.34 (0.82–2.20)	0.241		
Stroke	0.78 (0.40–1.51)	0.456		
Neoplasm	1.21 (0.56–2.62)	0.635		
Albumin	0.39 (0.24–0.62)	< 0.001	0.48 (0.27–0.84)	0.010
Hemoglobin	0.82 (0.74–0.92)	< 0.001	0.94 (0.83–1.07)	0.371
eGFR	0.99 (0.97–1.00)	0.055	0.99 (0.98–1.01)	0.275
BNP	1.00 (1.00–1.01)	0.111		
CRP	1.06 (0.94–1.18)	0.355		
Antiplatelet drugs	1.06 (0.66–1.70)	0.805		
DOAC	0.63 (0.41–0.97)	0.035	0.71 (0.45–1.13)	0.146
ACEi/ARB	0.91 (0.57–1.44)	0.676		
Beta-blocker	0.73 (0.46–1.17)	0.190		
MRA	1.36 (0.88–2.11)	0.167		

*ACEi* angiotensin converting enzyme inhibitor, *ARB* angiotensin receptor blocker, *BMI* body mass index, *BNP* brain natriuretic peptide, *CAD* coronary artery disease, *CI* confidence interval, *COPD* chronic obstructive pulmonary disease, *CRP* C-reactive protein, *DOAC* direct oral anticoagulant, *eGFR* estimated glomerular filtration rate, *HR* hazard ratio, *MRA* mineralocorticoid receptor antagonist

**Fig. 3:**
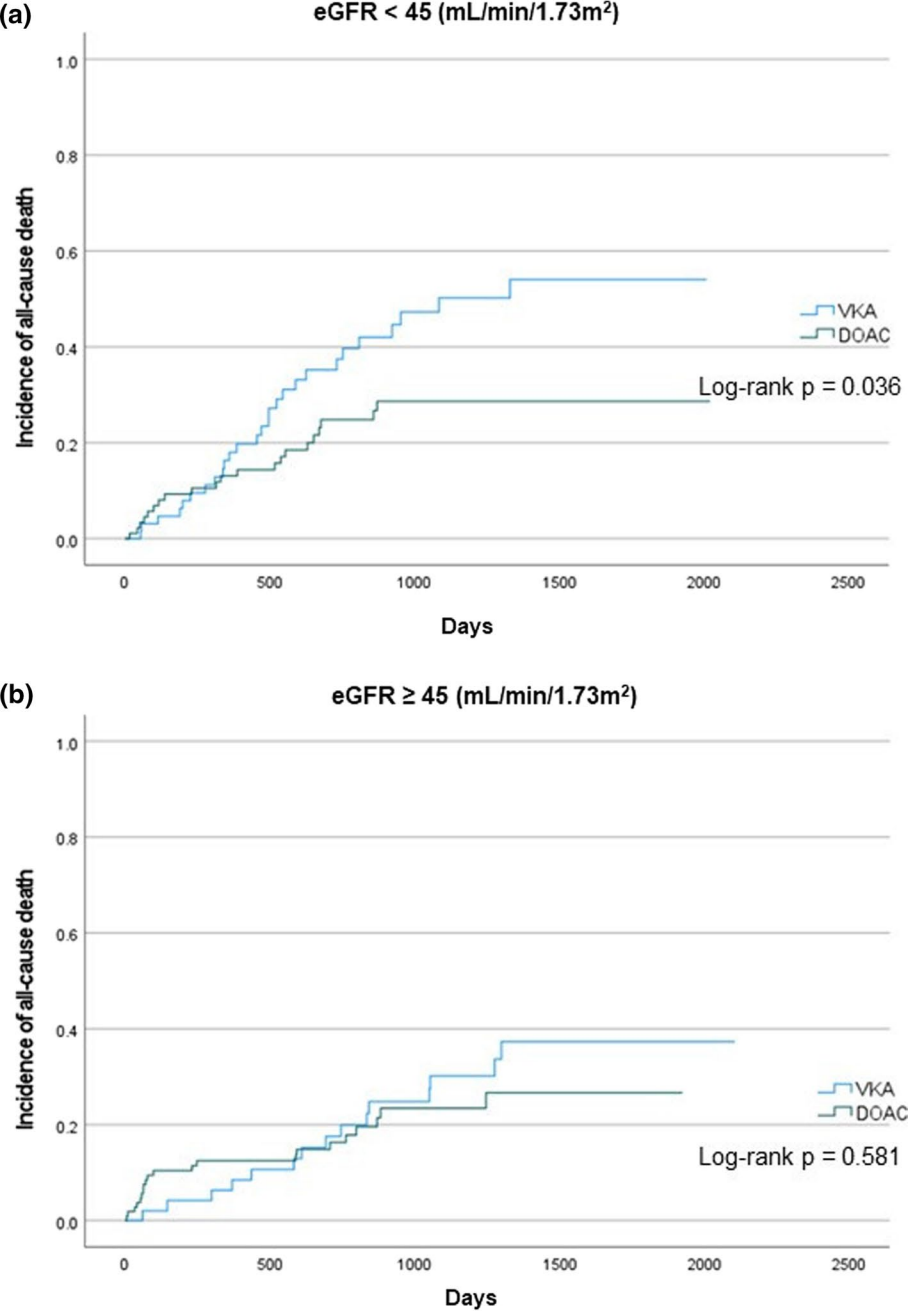
Kaplan–Meier analysis for the incidence of all-cause mortality in eGFR < 45 mL/min/1.73 m^2^ subgroup and eGFR ≥ 45 mL/min/1.73 m^2^ subgroup. DOAC administration was associated with lower mortality rates in the eGFR < 45 mL/min/1.73 m^2^ subgroup but not associated in the eGFR ≥ 45 mL/min/1.73 m^2^ subgroup. *DOAC* direct oral anticoagulant, *eGFR* estimated glomerular filtration rate, *VKA* vitamin K antagonists

The subgroup analysis for renal dysfunction is shown in Fig. 3. DOAC administration was associated with lower mortality rates in the eGFR < 45 mL/min/1.73 m^2^ subgroup (log-rank *p* = 0.036) but not in the eGFR ≥ 45 mL/min/1.73 m^2^ subgroup (log-rank *p* = 0.581). The Cox proportional hazard analysis revealed that DOAC therapy was independently associated with lower mortality after adjustments for age, diabetes mellitus, and albumin level (HR, 0.55; 95% CI 0.30–0.99; *p* = 0.045) in the eGFR < 45 mL/min/1.73 m^2^ subgroup (Table 4) but not associated in the eGFR ≥ 45 mL/min/1.73 m^2^ subgroup (HR, 1.27; 95% CI 0.60–2.67; *p* = 0.533).

**Table 4:** Cox multivariate analysis for all-cause death in subgroups

	eGFR < 45 (mL/min/1.73 m^2^)				eGFR ≥ 45 (mL/min/1.73 m^2^)			
	Unadjusted HR (95% CI)	*p* value	Adjusted HR (95% CI)	*p* value	Unadjusted HR (95% CI)	*p* value	Adjusted HR (95% CI)	*p* value
Age	1.05 (0.99–1.10)	0.076	1.03 (0.97–1.09)	0.346	1.08 (1.03–1.13)	0.001	1.04 (0.99–1.10)	0.165
Female	0.85 (0.48–1.50)	0.570			0.51 (0.25–1.01)	0.054	0.50 (0.22–1.11)	0.088
BMI	0.96 (0.89–1.03)	0.258			0.77 (0.68–0.87)	< 0.001	0.78 (0.68–0.90)	< 0.001
Hypertension	0.83 (0.45–1.55)	0.565			0.75 (0.39–1.44)	0.390		
Dyslipidemia	1.02 (0.55–1.87)	0.962			0.69 (0.29–1.66)	0.406		
Diabetes mellitus	1.71 (0.95–3.07)	0.072	1.83 (0.98–3.40)	0.057	0.79 (0.37–1.68)	0.537		
Hyperuricemia	1.50 (0.79–3.72)	0.221			0.88 (0.31–2.48)	0.801		
COPD	1.67 (0.52–5.39)	0.388			3.04 (1.33–6.95)	0.008	1.86 (0.64–5.37)	0.255
CAD	1.30 (0.69–2.45)	0.427			1.32 (0.60–2.89)	0.492		
Stroke	0.70 (0.30–1.65)	0.414			0.84 (0.30–2.39)	0.750		
Neoplasm	1.39 (0.50–3.89)	0.529			1.07 (0.33–3.50)	0.908		
Albumin	0.49 (0.26–0.93)	0.028	0.49 (0.25–0.95)	0.036	0.30 (0.15–0.59)	< 0.001	0.50 (0.22–1.12)	0.091
Hemoglobin	0.89 (0.76–1.04)	0.131			0.78 (0.67–0.93)	0.004	0.90 (0.73–1.11)	0.340
eGFR	0.99 (0.95–1.02)	0.429			0.99 (0.97–1.02)	0.748		
BNP	1.00 (1.00–1.01)	0.109			1.00 (1.00–1.01)	0.443		
CRP	0.96 (0.77–1.20)	0.707			1.25 (1.03–1.52)	0.026	1.20 (0.94–1.54)	0.153
Antiplatelet drugs	0.92 (0.50–1.72)	0.803			1.20 (0.58–2.48)	0.629		
DOAC	0.54 (0.30–0.95)	0.036	0.55 (0.30–0.99)	0.045	0.83 (0.43–1.61)	0.580	1.27 (0.60–2.67)	0.533
ACEi/ARB	1.27 (0.68–2.37)	0.448			0.63 (0.31–1.25)	0.185		
Beta-blocker	0.77 (0.42–1.44)	0.420			0.70 (0.34–1.42)	0.320		
MRA	1.20 (0.68–2.13)	0.522			1.75 (0.86–3.57)	0.121		

*ACEi* angiotensin converting enzyme inhibitor, *ARB* angiotensin receptor blocker, *BMI* body mass index, *BNP* brain natriuretic peptide, *CAD* coronary artery disease, *CI* confidence interval, *COPD* chronic obstructive pulmonary disease, *CRP* C-reactive protein, *DOAC* direct oral anticoagulant, *eGFR* estimated glomerular filtration rate, *HR* hazard ratio, *MRA* mineralocorticoid receptor antagonist

## Discussion

The major finding of our study was that DOAC therapy was independently associated with lower mortality rate in patients with renal dysfunction in the Japanese population. Previous studies showed that use of DOACs was associated with lower risks of stroke and systemic embolism, but its association with all-cause mortality has not been fully evaluated especially in patients with renal dysfunction. Four mega studies were conducted for each DOAC; however, three studies (ROCKET AF, RE-LY [Randomized Evaluation of Long-term Anticoagulant Therapy], and ENGAGE AF-TIMI 48 [Effective Anticoagulation with Factor xA Next Generation in Atrial Fibrillation–Thrombolysis in Myocardial Infarction study 48]) excluded patients with creatinine clearance values < 30 mL/min. The ARISTOTLE (Apixaban for Reduction in Stroke and Other Thromboembolic Events in Atrial Fibrillation) trial excluded patients with creatinine clearance values < 25 mL/min. Recently, Makani et al. reported the safety and efficacy of DOAC use in patients with concomitant renal dysfunction and AF. In this study, the patients were classified into three groups according to eGFR (> 60, 30–60, and < 30 mL/min/1.73 m^2^), and DOAC administration was associated with lower risk of mortality across all stages of renal dysfunction [9]. However, the efficacy of DOACs in the patients with renal dysfunction was not equal across races and ethnicity because the meta-analysis of five mega studies revealed that the usual DOAC dose as compared with that of VKA was effective for the prevention of stroke or systemic prevention and safer in terms of reducing the risk of major bleeding in Asians than in non-Asians [10]. The difference in the efficacy of DOACs between the populations remains an unsolved problem.

In the Japanese population, three major observational studies were conducted in patients with AF [13–15]. The J-RHYTHM Registry 2, a multicenter observational study, revealed that the incidence of all-cause death in the DOAC group was extremely low as compared with those in the non-OAC and VKA groups. However, this study expanded the follow-up period in the J-RHYTHM Registry, which enrolled patients from January 2010 to July 2010. At baseline, the antithrombotic therapy was VKA only (5737 patients), but 923 patients were switched from VKA to DOAC, and the exact follow-up time after the switch was unknown. The Fushimi AF registry was a prospective, observational multicenter cohort study conducted between March 2011 and November 2015. In this registry, DOAC use was not statistically associated with stroke or systemin embolism (*p* = 0.70) or major bleeding (*p* = 0.34) after propensity score matching, but all-cause mortality was not evaluated [15]. The Shinken Database is a single-hospital-based cohort that was started in 2004, and the 9-year trends of anticoagulation therapy and thromboembolic events or major bleeding were analyzed, but the incidence of all-cause death was not evaluated.

Recently, the SAKURA-AF registry was established to compare the safety and efficacy of DOACs and VKAs in the Japanese AF population and revealed that the incidence rates of stroke/systemic embolism, major bleeding, and all-cause mortality were not statistically significantly different between the VKA and DOAC groups after adjustment by propensity score matching.

Our study represents a real-world analysis of the efficacy of DOACs in elderly NVAF patients with renal dysfunction. As compared with VKA administration, the use of DOACs was associated with lower mortality rates among patients with AF and renal dysfunction. Although this was a retrospective analysis, our findings suggest that DOACs do not appear to be inferior to VKA, and physicians should not hesitate to prescribe DOACs for patients with concomitant renal dysfunction and AF.

Two hypotheses may explain the association of DOAC use with the lower risk of mortality in our registry. First, the SAKURA-AF registry included TTR, which was relatively high (65.4% ± 31.1%) as compared with those in other recent randomized controlled trials (mean TTR: 55–68%) [16–18]. The better TTR in the VKA users would result in a low clinical event rate as in the DOAC users. Although our registry did not include TTR, it would be lower in the eGFR < 45 mL/min/1.73 m^2^ subgroup than in the eGFR ≥ 45 mL/min/1.73 m^2^ subgroup because renal dysfunction was identified as risk factor of lower TTR [6]. Moreover, Lin et al. reported that VKA was associated with higher incident of significant bleeding and myocardial infarction in patients with stage 3–5 CKD [19]. Second, the mean CHADS2 score in our registry was 3.0 in both subgroups, which was relatively higher than those in other registries in Japan, ranging from 1.8 to 2.1 [15, 20, 21], or the SAKURA-AF registry, ranging from 1.7 to 1.9 [22]. Patients with high CHADS2 scores have more comorbidities and are associated with the use of more concomitant drug treatments, that is, polypharmacy. A previous study reported that adverse clinical outcomes such as death and bleeding complications occurred more frequently in patients with polypharmacy [23–25]. These outcomes could be derived from the increasing risk of drug-to-drug interactions with the use of many concomitant drug treatments. Although our registry did not analyze the number of drugs used, having more comorbidities would be associated with the incidence of stroke or other adverse clinical events and lead to high mortality in patient with renal dysfunction. Further randomized control trials are needed to certify our hypotheses.

### Limitations

This study has several limitations. First, the sample size was relatively small and it would not be enough to represent the general population. Second, we did not evaluate TTR in the VKA group. Third, we did not specify the kinds of DOACs used. In the 2019 AHA/ACC guideline, only VKA and apixaban are recommended for patients with severe CKD, and other DOACs are not recommended [26]. Safety and efficacy would differ between DOACs. Fourth, the HAS-BLED (hypertension, abnormal renal/liver function, previous stroke, bleeding history or predisposition, labile international normalized ratio [INR], elderly and drugs/alcohol consumption) score could not be determined because information on alcohol dependency was unavailable. Fifth, only three patients had severe renal dysfunction (eGFR < 15 mL/min/1.73 m^2^), and we could not perform a sufficient evaluation for these patients.

## Conclusion

DOAC therapy for HF patients with NVAF and renal dysfunction is associated with lower mortality than VKA therapy.
